# Malaria transmission modelling: a network perspective

**DOI:** 10.1186/2049-9957-1-11

**Published:** 2012-11-01

**Authors:** Jiming Liu, Bo Yang, William K Cheung, Guojing Yang

**Affiliations:** 1 Department of Computer Science, Hong Kong Baptist University, Kowloon Tong, Hong Kong; 2 College of Computer Science and Technology, Jilin University, Changchun, China; 3 The Jockey Club School of Public Health and Primary Care, Chinese University of Hong Kong, Hong Kong

## Abstract

Malaria transmission can be affected by multiple or even hidden factors, making it difficult to timely and accurately predict the impact of elimination and eradication programs that have been undertaken and the potential resurgence and spread that may continue to emerge. One approach at the moment is to develop and deploy surveillance systems in an attempt to identify them as timely as possible and thus to enable policy makers to modify and implement strategies for further preventing the transmission. Most of the surveillance data will be of temporal and spatial nature. From an interdisciplinary point of view, it would be interesting to ask the following important as well as challenging question: Based on the available surveillance data in temporal and spatial forms, how can we build a more effective surveillance mechanism for monitoring and early detecting the relative prevalence and transmission patterns of malaria? What we can note from the existing clustering-based surveillance software systems is that they do not infer the underlying *transmission networks* of malaria. However, such networks can be quite informative and insightful as they characterize how malaria transmits from one place to another. They can also in turn allow public health policy makers and researchers to uncover the hidden and interacting factors such as environment, genetics and ecology and to discover/predict malaria transmission patterns/trends. The network perspective further extends the present approaches to modelling malaria transmission based on a set of chosen factors. In this article, we survey the related work on transmission network inference, discuss how such an approach can be utilized in developing an effective computational means for inferring malaria transmission networks based on partial surveillance data, and what methodological steps and issues may be involved in its formulation and validation.

## Multilingual abstracts

Please see Additional file 1 for translations of the abstract into the six official working languages of the United Nations.

## Background

Malaria transmission is challenging to model; its vector can be quite complex due to topographical and climatic variations as well as human mobility [1]. One of the United Nations (UN) Millennium Development Goals is to “have halted by 2015 and begun to reverse the incidence of malaria” which annually causes ~1 million death or >1 death every 30–60 second [2,3]. World Health Organization (WHO) has suggested that the most important measure is a timely response with the implementation of effective interventions once it has been detected [4]. This requires an effective monitoring/surveillance system that can provide long range forecasting, early warning, and early detection [5]. Towards this end, malaria transmission patterns will be informative in performing such surveillance functions.

This article provides a review of related work on how to develop a computational means for inferring malaria transmission networks in populations, which incorporates: (1) partial surveillance data over time, i.e., the temporal-spatial distributions of cases of infection, and (2) infection models of malaria. A transmission network to characterize the temporal-spatial patterns of disease transmission, or a temporal-spatial disease transmission network, consists of a set of nodes and a set of links that connect them, where the nodes correspond to spatial locations, such as villages, with reported/observed disease incidences over time, and the directional links connecting the nodes correspond to the probability/likelihood of disease “transmission” from one node to another over time, e.g., hidden pathways of malaria transmission.

Technically, the problem of computationally inferring malaria transmission networks is both interesting and challenging because, during the process of disease spread, the reported infection cases do not directly reflect the full extent of transmission, nor the underlying transmission patterns. It would be desirable for us to detect such networks from the partially available surveillance data. In doing so, we may incorporate a malaria infection model, e.g., the Ross-MacDonald model [6].

In this article, we discuss how such a computational method differs from existing methods of network inference, in the light of the unique nature of malaria transmission dynamics. In computer science, related studies have been carried out to solve the problem of inferring information diffusion networks from Web data [7-10]. These studies only consider temporal information and cannot readily be extended sufficiently to incorporate additional information, such as spatial, environmental, climatic, and clinical information. Also, most of the methods are based on independent cascading models, assuming that one node will be independently infected by others with respective probabilities, and cannot readily integrate more complicated infection/propagation models.

## Methods

We searched and reviewed the related research papers in (1) bibliographical databases including Web of Science and PubMed, (2) international conferences including *ACM-SIGKDD*, *ICDM*, *ICML*, *SIAM-SDM*, *WWW*, etc., and (3) World Health Organization (WHO) reports. The aim of our survey is to find and study (1) existing methods for modelling disease infections and epidemics-like transmission processes based on structural representations such as transmission paths or networks, and furthermore (2) those for inferring the underlying transmission networks based on temporal and/or spatial surveillance data.

### Scan statistics-based clustering and network-based epidemic dynamics modelling

We first examined existing studies on modelling temporal-spatial patterns of epidemic dynamics. We started by evaluating the scan statistics-based clustering methods and their related software tools for modelling malaria transmission (which are also useful for detecting active foci or hotspots over time and space). Our survey aims to identify the need for a more explicitly structured representation of disease spread, e.g., the interrelationships among different locations due to the heterogeneous temporal-spatial factors affecting hosts, vectors, and parasites at various scales. Such a representation would be particularly desired in planning cost-effective intervention strategies. We then surveyed the related studies in both epidemiology and other disciplines that had demonstrated how disease spread and/or information dynamics could be revealed based on network representations, i.e., disease spread dynamics on networks. In doing so, we focused on how dynamics may vary with respect to the characteristics of networks, e.g., regular, small-world, or scale-free networks, as well as human behaviour, e.g., mobility.

### Inferring networks from temporal data

Once we confirmed the role of networks in understanding disease spread dynamics, our next logical step was to investigate how existing studies had attempted to predict the structures of underlying transmission networks, whether indirectly, e.g., through information on human mobility and social contact activities, or directly, e.g., based on observed/reported cases of infection. We paid special attention to the present methods that had been implemented for the purpose of inferring an underlying transmission network (links) from its observed (node) activities. The surveyed work, although may have appeared in the fields other than epidemiology, would provide us with a good understanding of the general methodology for detecting interaction networks from observations over time and space.

## Findings and discussion

**Figure 1:**
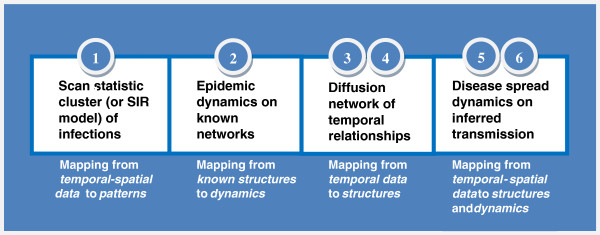
**The four approaches (as shown in the four boxes) discussed in this article are listed by highlighting their distinct characteristics.** The labelled numbers correspond to the section numbers in Table 1.

The key concepts, their corresponding representative examples, and publications that we reviewed are summarized in Table 1. As depicted in Figure 1, they constitute four distinct approaches. In what follows, we will present our findings and observations.

**Table 1:** A summary of key concepts, representative examples, and corresponding references

**Key concepts and considerations**	**Representative examples**	**References**
1. Temporal-spatial characterization	Scan statistics-based clustering	[11]
	Scan software tools	[12-15]
	Other applications (active foci or hotspots)	[16]
Related factors	Biology, environment, and socio-economy affecting interactions among hosts, vectors, and parasites at various scales	[17-19]
	Entomological inoculation rates, vector capacity, or force of infection	[20]
	A combination of epidemiological, geographical, and demographic factors	[21]
2. Modelling disease and/or information dynamics on networks	Dynamics of infectious diseases on regular, small-world, or scale-free networks	[22-27]
	Critical value analysis of typical epidemics on complex network	[28-33]
	Diffusion of rumours or innovation on social networks	[34-36]
	Viral marketing and recommendation strategies	[37-39]
	Cascading in virtual blog spaces, and their propagation trends	[10,40-43]
Related factors	Alternative spatial representations	[44]
	Effects of human mobility on the dynamics of disease transmission	[45]
3. Understanding the structures of underlying transmission networks via indirect means	Population travelling and mobility patterns	[46,47]
	Social contact activities	[48-50]
	Sexual relationships	[51]
4. Inferring transmission parameters from data	EM-based estimation algorithm to infer daily transmission rate between households	[52]
	Markov Chain Monte Carlo (MCMC) method to estimate transmission parameters	[53]
5. Inferring an underlying network from data	Social networks based on the interpersonal interaction records	[54-58]
	Interaction networks between proteins in a cell	[59,60]
	Supervised classification	[7]
	Expectation-maximization (EM)-like algorithm	[10]
	Narrow and deep tree-like structure analysis	[8]
	Likelihood-maximization	[9]
	Independent cascading models	[41]
6. Computational issues	Conventional optimization methods	[61]
	Potentially large-scale and/or dynamically-evolving surveillance data, e.g., over decades of temporal intervals	[62-64]
	Different levels of spatial categories	[62,63]
	Multiple environmental or biological factors incorporated	[19,64]
	Alternative AOC methods	[65-67]

### Scan statistics-based clustering and network-based epidemic dynamics modeling

Malaria transmission can be affected by multiple factors, such as biology, environment, and socio-economy, that directly impinge on the interactions among hosts, vectors, and parasites at varying degrees and scales [17-19]. A feasible means to model malaria transmission is to rely on passive case reporting surveillance systems or surveys through local, national, or regional public health and medical organizations. Most of the data collected from such systems or surveys may contain temporal, spatial, clinical, and/or demographic information, and may cover only arbitrary locations and age-groups. At the moment, temporal-spatial scan statistics-based clustering techniques have been applied to the analysis and characterization of temporal-spatial patterns of malaria [11]. In doing so, software tools [12-15] have been used to manage and geographically map reported malaria cases, and to test whether the cases are randomly or significantly distributed with spatial or space-time disease clusters. Sometimes, for a more accurate malaria map, additional information may be incorporated, e.g., by means of modelling entomological inoculation rates, vector capacity, or force of infection [20], or by using a combination of epidemiological, geographical, and demographic data [21]. Generally speaking, such temporal-spatial techniques do not infer the underlying transmission patterns (modelled as *networks*) of malaria. Such transmission networks can be informative and perceptive, as they characterize how malaria is diffused or transmitted from one location to another (e.g., across villages) over time, providing a new way of detecting the active foci or hotspots of malaria transmission other than directly estimating epidemiological factors or identifying clusters with above-average transmission intensity [16]. Therefore, by integrating both temporal-spatial clusters of cases of infection and temporal-spatial transmission networks of malaria, existing local, national, or regional surveillance systems can further be enhanced in their functional capacities of predicting and analyzing the impact of malaria transmission and their underlying factors, as well as evaluating existing intervention or eradication strategies and guiding new control efforts.

Next, we survey related studies on modelling the dynamics of epidemics on networks. Although the subjects involved in different epidemics can vary considerably, many can be modelled by either SIR/SIS models [68-70], cascading models [37], or threshold models [71,72]. The assumption behind the basic SIR or SIS models is that an individual in a population will be in one of the three states: suspected (S), infected (I), and recovered (R). If individuals are viewed as nodes, and the contacts between them as links, a network can be obtained that describes who will infect whom with what probabilities based on the SIR or SIS model. Grassberger first studied the dynamics of infectious diseases that propagate on regular networks using the percolation theory [22]. Studies have revealed that many real-world networks, including social networks in which infectious diseases propagate [26,27], are either small-world [23] or scale-free [24,25], rather than regular or random, as thought previously [73].

As the underlying structures of networks will influence the effect that dynamics of epidemics will have on them, researchers, such as Pastor-Satorras and Vespignani, have made several contributions to critical value analysis of typical epidemics on different types of complex network [28-33]. Based on the mean-field theory, they found that, compared with homogeneous networks, scale-free networks are fragile to the invasion of infectious diseases, computer viruses, or any other type of epidemics. In addition, researchers have also considered different spatial representations in modelling directly transmitted infectious diseases [44] and the effects of human mobility on the dynamics of disease transmission on networks [45].

Epidemics on networks have also been studied in various disciplines. Sociologists are concerned with the diffusion of rumours or innovation on social networks [34-36]; economists have studied viral marketing and recommendation strategies by considering cascading dynamics as well as the network effects of vital nodes [37-39]; computer scientists are interested in how some topics can quickly cascade in virtual blog spaces, and their propagation trends [10,40,42,43].

As can be noted, existing studies have contributed to the modelling of disease and/or information dynamics on networks. However, most of them have made a strong assumption that the structures of underlying networks, over which a disease spreads, are *known beforehand*. This may not hold in the real world, as the structures allowing the underlying transmission networks to emerge will not be known directly (even though in some cases, indirect means have been used, e.g., based on population travelling and mobility patterns [46,47], social contact activities [48-50], or sexual relationships [51]); what may be obtained is only the time when particular nodes are found infected, but not how they get infected, nor how they affect their neighbouring areas. Then, the question that remains unanswered is how to model disease spread dynamics on hidden networks, which in turn leads to the basic question of how to computationally infer such hidden transmission networks based on partial temporal-spatial surveillance data.

### Inferring networks from temporal data

Inferring underlying transmission and propagation processes by analyzing the observable spreading patterns has drawn special attention from several domains, ranging from the path inference of information propagation [7,9,54], the construction of social networks based on the interpersonal interaction records [55-58], to the inference of interaction networks between proteins in a cell [59,60]. Adar and Adamic [7] first studied the problem of inferring information dynamics in blog space, and formulated the diffusion-network prediction problem as a supervised classification problem. Gruhl et al. [10] developed a method for inferring how topics spread among the blogs and proposed an expectation-maximization (EM)-like algorithm for estimating propagation probabilities over an underlying diffusion network. Liben-Nowell and Kleinberg [8] studied information flow on a global scale by analyzing chain-letter datasets, and indicated that the propagations of chain letters spread on social networks are shaped by narrow and deep tree-like structures with most one-child nodes. Gomez-Rodriguez et al. [9,74] formulated the problem of inferring a diffusion network into a likelihood-maximization task, and proposed a greedy algorithm that can approximately learn underlying networks from observed infection sequences.

In addition to the above, a few studies have been carried out in the domain of epidemiology. Zelner et al. [52] examined how infections spread after the point-source outbreaks of secondary norovirus transmission between households by analyzing a real-world dataset that described a food-borne norovirus outbreak in 30 daycare centres in Stockholm. They extended the basic SIR model by explicitly considering the incubation period of norovirus, and correspondingly proposed an EM-based estimation algorithm to infer daily transmission rate, and the means and shapes of the Poisson distributions of incubation and infectious periods. Similarly, Hohle et al. [53] proposed an inference technique based on the Markov Chain Monte Carlo (MCMC) method to estimate transmission parameters for an infectious disease from time series data by allowing variability in the incubation period.

To date, most of the existing work has mainly focused on how to use temporal information to infer underlying transmission networks, but neglected spatial information, which would be equally critical in shaping the underlying networks. To the best of our knowledge, almost none of them have explicitly studied the problem of designing a surveillance mechanism based on the idea of *inferring disease transmission networks and corresponding dynamics from collected data* through the integration of both temporal and spatial information.

### Towards network-based malaria transmission modelling

Further to the above review of the related work, we next discuss a potential methodology for developing a computational means of inferring disease transmission networks based on partial temporal-spatial surveillance data.

### Inferring transmission networks from temporal-spatial surveillance data

First of all, it should be pointed out that although the idea of inferring the underlying networks of disease transmission from surveillance data can benefit from the related methods in computer science [7-10,74], the former differs from the latter in some fundamental ways, in the light of the unique nature of disease transmission dynamics:

(1) The existing methods mainly focus on learning information diffusion networks from Web data, and thus when inferring the structures of diffusion networks, most of them merely consider temporal information, and neglect spatial, environmental, climatic, demographic, clinical, and other key factors as related to malaria transmission.

(2) Most of them are based on independent cascading models [7-9,25,41,43,74] that are widely used to characterize information flows, with the assumption that one node will be independently infected by others with respective probabilities. However, in the real world, infectious diseases follow much more complicated infection/propagation models such as the ones of malaria transmission [75]. Reasonable transmission networks may be discovered only when their corresponding infection/propagation models are considered sufficiently during the course of formulation and computation.

In view of the above, it is clear that there exists a need for developing novel methods for modelling and inferring disease transmission networks from surveillance data that would enable us to incorporate temporal, spatial, as well as other recorded attributes. To do so, we need to design corresponding algorithms. An illustrative example of the probabilistic approach to modelling and inferring malaria transmission networks is given as follows.

Suppose that surveillance data on malaria consists of collected case observations that are formatted as *M* temporal-spatial series (corresponding to *M* locations, such as villages). Items in such a temporal-spatial series take the form of 4-tuple (*i*, *t*_*i*_, *k*_*i*_, *attr*_*i*_), which indicates that *k*_*i*_ cases at node *i* with an attribute *attr*_*i*_ are reported/observed during a time span *t*_*i*_. Note that node *i* corresponds to a specific location, such as a village, whose geographic, environmental, climatic, demographic, or clinical attributes can be incorporated in *attr*_*i*_. *t*_*i*_ may be measured in different scales, such as daily, weekly, or monthly. A probabilistic approach to network-based disease transmission modelling can readily be carried out based on the following steps:

Step 1. Let *λ* be the infectious strength, which indicates how likely one node becomes infected by its neighbours, or in other words, how likely a disease propagates to one node from its neighbours. Drawing on the basic Ross-MacDonald model, the infectious strength of malaria at node *i* during an interval *t*_*i*_ (i.e., a disease infection/propagation model integrating geographic, environmental, climatic, demographic, and clinical information) is defined as:

$$\lambda \left(B_{i},t_{i},k_{i},attr_{i}\right)=\left(a^{2}bcmt_{i}/\mu \right)\times \sum_{j\in B_{i}}k_{j}$$

where *B*_*i*_ denotes the neighbours of node *i* in network *N*. 1/*μ* denotes the life expectancy of mosquitoes, and *m* corresponds to the number of mosquitoes per human host in location *i*. *a*, *b*, and *c* indicate the rate of biting humans by a single mosquito, the proportion of infected bites in humans that produce an infection, and the transmission efficiency from humans to mosquitoes, respectively, which are empirically/clinically determined for the endemic areas and populations in focus. $\sum_{j\in B_{i}}k_{j}$ denotes the total number of the infected cases within the neighbouring locations of node *i*.

Step 2. Compute the likelihood of the time series *r* being observed as follows:

$$\begin{array}{l} L_{r}\left(N|\lambda \right)=\prod_{i=1}^{l_{r}}P\left(t_{i}<X_{i}\leq t_{i}+\Delta t\right)\\ \;=\prod_{i=1}^{l_{r}}\lambda \left(B_{i},t_{i},k_{i},attr_{i}\right)e^{-\lambda \left(B_{i},t_{i},k_{i},attr_{i}\right)t_{i}} \end{array}$$

where *N* denotes an underlying transmission network*,* on which the disease spreads according to a disease-specific model *λ*, and *l*_*r*_ is the length of the temporal-spatial series *r*.

Step 3. Given *M* temporal-spatial series observed and an infection/propagation model *λ*, define and maximize an objective function based on the likelihood function and a penalty term to avoid over-fitting that happens when the model’s degree of freedom is too higher with respect to the number of observed data:

$$O\left(N\right)=L\left(N|\lambda \right)+||N|{|}_{L_{1}}$$

where *L*(*N*|*λ*) = ∏ _*r* = 1_^*M*^*L*_*r*_(*N*|*λ*) and $||N|{|}_{L_{1}}$ is the *L*_1_-norm term of the adjacency matrix of network *N*.

The optimization can be carried out by, for example, formulating *O*(*N*) such that it is a submodular function with the property of diminishing return [63], and then using the submodular greedy method to determine an approximately optimal transmission network. To handle the case of network inference that potentially involves large-scale and/or dynamically-evolving surveillance data, e.g., over decades of temporal intervals [19,64,65], thousands of locations in different levels of spatial categories [64,65], and multiple environmental or biological factors considered [65,66], alternative optimization methods can be considered; one of the possible ways is to utilize *autonomy-oriented computing* (AOC) [15,27,67]. One can take further advantage of the autonomy aspect emphasized in the AOC paradigm and consider that some active surveillance strategies can be employed. Then, the optimization space will not span only the model parameter space but also the allowable surveillance strategy space so as to further boost to the accuracy of the transmission network inference.

To summarize, adopting such a network approach can be quite useful for public health authorities and epidemiologists to gain insights into the impacts of disease spread over time and space and the underlying factors (e.g., corresponding environmental/climatic factors, mosquito ecology/genetic evolution, human mobility, and control/intervention strategies) in a region, e.g., through constructing and comparing a series of transmission networks.

### Empirical evaluation and application

Finally, we comment on how to validate the method of inferring malaria transmission networks, particularly by testing it based on the available malaria dataset historically collected from various endemic areas.

We may validate the network-based modelling method by means of a cross-validation strategy. In doing so, we divide the dataset into two parts. The first part is used to determine the transmission network covering the area in focus. Based on the inferred transmission network and a malaria infection model, we simulate malaria transmission in the respective region based on the following steps: (1) initialize the onset of malaria propagation on the detected transmission network by selecting the nodes corresponding to the earlier reported cases in the real data; (2) in each iteration (each iteration corresponds to a time scale, e.g., weekly), each infected node tries to infect its neighbours (determined by the transmission network); (3) record the obtained temporal-spatial distribution (times are denoted by the sequence of iterations and locations are denoted by the geographical attributes of nodes) of infected cases during the whole simulation for the covered period. Then, we compare the cases sampled by the aforementioned simulation with the real cases provided by the second part of the dataset, and evaluate the utility of the inferred network in terms of some defined metrics. The divisions of the dataset could be performed using different ratios of learning data and testing data.

## Conclusion

In view of the need for healthcare providers, government disease control and prevention organizations, and international agencies to predict and analyze the impacts of malaria transmission and their underlying factors, in this article we have discussed various approaches related to epidemic dynamics modelling and transmission mechanism/network inference from surveillance data, and have pointed out the needs for future research (see future research priorities in Table 2). In particular, we have addressed how to extend the existing computational concepts and methods of transmission networks to those of inferring and predicting the prevalence and transmission patterns of malaria in populations, and have outlined a potential methodology that would enable us to develop and apply a computational method to automatically infer the underlying malaria transmission network that utilizes (1) partial surveillance data, i.e., the temporal-spatial distributions of cases of infection, and (2) infection/propagation models of the disease.

**Table 2:** Future research priorities in network-based malaria transmission modelling

	
Inference of transmission networks	· Incorporating partial surveillance data over time, i.e., the temporal-spatial distributions of cases of infection
	· Constructing specific infection models of malaria, while incorporating additional information, such as geographic, environmental, climatic, demographic, clinical, and behavioural information
	· Developing computational tractable probabilistic methods, as well as extending the existing models proposed in computer science (e.g., independent cascading models)
Use of transmission networks	· Validating inferred transmission networks by testing them with available malaria data
	· Predicting and analyzing the impact of malaria transmission and their underlying factors over time and space through constructing and comparing a series of transmission networks
	· Evaluating existing intervention or eradication strategies and guiding new control efforts

## Competing interests

The authors declare that they have no competing interests.

## Authors’ contributions

JL and BY designed research; JL, BY, WKC and GY performed research; JL and BY wrote the paper; All authors read and approved the final manuscript.

## Supplementary Material

Additional file 1 Multilingual abstracts in the six official working languages of the United Nations. Click here for file
