# Supplementary material for: Malaria transmission modelling: a network perspective
Source: Infect Dis Poverty. 2012 Nov 1;1:11. doi: 10.1186/2049-9957-1-11 (PMC3710080; doi:10.1186/2049-9957-1-11)
Supplement: Additional file 1 — Multilingual abstracts in the six official working languages of the United Nations. [file 2049-9957-1-11-S1.pdf]

Translation of the abstract into the six official working languages of the United Nations

نموذج نقل الملاريا: من منظور شبكة الانترنت

جىمينغ ليو, بو يانغ ، وليام تشيونغ وجيوجينغ يانغ

ملخص
يمكن أن يتأثر انتقال الملاريا بعوامل متعددة أو حتى مخفية، مما يجعل من الصعب التنبؤ بدقة و في الوقت
المناسب بتأثير برامج القضاء على و الازالة التي تم الاضطلاع بها والعودة المحتملة والانتشار الذي قد يستمر في
يوجد نهج واحد في هذا الوقت هو تطوير ونشر أنظمة المراقبة في محاولة لتحديد الوقت المناسب قدر .الظهور
ان معظم .الامكان وبالتالي تمكين واضعي السياسات من تعديل وتنفيذ استراتيجيات لمنع زيادة انتقال العدوى
من وجهة النظر المتعددة التخصصات، سيكون من المثير . البيانات تكون ذات طبيعة مكانية و زمانية مؤقتة
للاهتمام أن نسأل السؤال التالي الهام والصعب: استنادا إلى البيانات المتاحة لأشكال المراقبة الزمانية المؤقتة
والمكانية، كيف يمكننا بناء آلية أكثر فعالية لمراقبة الرصد والكشف المبكر النسبي لانتشار وانتقال أنماط الملاريا؟
ما يمكننا ملاحظته من القائمة التي تجمع الأنظمة المستندة إلى برامج مراقبة هو أنها لا تظهر شبكات النقل
ومع ذلك، يمكن أن تكون هذه الشبكات مصدرا جيدا جدا و نافذا للمعلومات فهي تميز كيف .الضمنية للملاريا
فإنه يمكننا بدورها أن تسمح أيضا لواضعي السياسات الصحية العامة والباحثين .تنقل الملاريا من مكان إلى آخر
التنبؤ بأنماط/ الاتجاهات / للكشف عن العوامل الخفية والمتداخلة مثل البيئة وعلم الوراثة وعلم البيئة واكتشاف
منظور الشبكة كذلك إلى المنهج الحالي للانتقال النموذجي للملاريا بالاعتماد على مجموعة يمتد انتقال الملاريا.
في هذه المقالة، نحن نقوم بدراسة الأعمال ذات الصلة على شبكة الانترنت الخاصة بالنقل .من العوامل المختارة
لغرض الاستدلال، ومناقشة كيف يمكن استخدام هذا النهج في تطوير وسيلة حاسوبية فعالة لاستنتاج انتقال الملاريا
استنادا إلى بيانات الإشراف الجزئي، وما هي الخطوات والمسائل المنهجية المشاركة في تقرير صياغتها والتحقق
.من صحتها

Translated from English version into Arabic by Morano El-Kholy, through

TRANSLATORS
WITHOUT BORDERS

疟疾传播的建模：网络视角

Liu Ji-ming, Yang Bo, William Cheung et Yang Guo-jing

摘要

疟疾的传播受多种乃至潜在因素的影响，因而对当前消灭根除疾病的工作效果以及可能持续出现的疾病复发和传播展开准确及时的预估造成了困难。现阶段的方法是设计和布置监测系统以期尽早的发现并确认病情，从而使决策者可以修改并实施其对策，进而阻止疾病的传播。大多数的监测数据具备时空属性。从交叉学科的角度而言，一个既有趣又充满挑战的问题是：鉴于可利用的时空监测数据，如何设计一种更加有效的监测机制，从而实现对疟疾流行传播模式的监控和早期发现。我们注意到现行的基于聚类的监测软件系统不能推演疟疾的潜在传播网络。然而，重要的是此类网络可以刻画疟疾从一地传播到另外一地，因而具备很强的信息性和洞察性。因此，它们可以被用以帮助公共卫生领域的决策者和研究者揭示那些隐含且相互作用的影响因素，诸如环境、遗传和生态，从而发现并预测疟疾传播的模式或趋势。网络分析可以进一步扩充现有基于选定因素的疟疾传播的建模方法。本文中，我们综述了推演传播网络的相关工作，讨论了如何应用此类方法设计一种有效的计算手段用以推演疟疾的传播网络，以及和其理论定式和实证研究相关的方法步骤和议题。

Translated from English version into Chinese by Liu Ji-ming

Modélisation de la Transmission du paludisme : Une Perspective de réseau

Liu Ji-ming, Yang Bo, William Cheung et Yang Guo-jing

Résumé

La transmission du paludisme peut être affectée par de multiples facteurs qui peuvent aussi être encore cachés, rendant difficile ,de manière rapide et efficace, prédire l'impact de l'élimination et l'éradication des programmes qui ont été entrepris et la résurgence et la propagation potentielle qui puissent continuer à émerger. Une approche à l'heure actuelle consiste à développer et déployer des systèmes de surveillance pour tenter de les identifier le plus tôt possible afin de permettre aux décideurs politiques de modifier et de mettre en œuvre des stratégies de prévention pour encore prévenir la transmission. La plupart des données de surveillance seront de nature temporelle et spatiale. D'un point de vue interdisciplinaire, il serait intéressant de poser cet importante et délicate question : Selon les données de surveillance disponibles sous formes temporelles et spatiales, comment peut-on construire un mécanisme de surveillance plus efficace et détecter très tôt les schémas relatives de prévalence et de transmission du paludisme ? Ce que nous pouvons observer des systèmes de surveillance existants, basés sur le groupage, c'est qu'ils n'indiquent pas les systèmes sous-jacents de transmission du paludisme. Cependant, ces réseaux peuvent être tout à fait instructifs et perspicaces puisqu'ils caractérisent comment le paludisme se transmet d'un endroit à l'autre. Ils peuvent aussi permettre à leur tour aux prescripteurs de politique de santé publique et les chercheurs de découvrir les facteurs cachés et interdépendants tels que l'écologie, la génétique et l'environnement et à découvrir/prédire les schémas de transmission du paludisme. La perspective de réseau étend a nouveau les approches actuelles à la modélisation de la transmission du paludisme basée sur un ensemble de facteurs choisis. Dans cet article, nous menons une enquête sur les travaux concernant l'extension des réseaux de transmission, discuter du comment une telle approche peut servir à l'élaboration d'un moyen efficace de calcul pour indiquer les systèmes de transmission du paludisme en se basant sur les données de surveillance partielle des réseaux ,

et quels sont les étapes méthodologiques et les problématiques qui pourraient intervenir dans sa formulation et sa validation.

Translated from English version into French by Nesrine Echroudi, through

TRANSLATORS
WITHOUT BORDERS

Моделирование передачи малярии: сетевой подход

Джиминг Лью, Бо Янг, Вильям Чеюнг и Гуоджинг Янг

Аннотация

На передачу малярии могут влиять многочисленные или даже скрытые факторы, затрудняя тем самым своевременный и точный прогноз результатов внедряемых программ по устранению и ликвидации этой болезни, а также потенциального повторного возникновения и распространения малярии. Одним из применяемых сейчас методов является разработка и развёртывание систем наблюдения для как можно более раннего выявления этих факторов, чтобы таким образом позволить должностным лицам изменять стратегии и применять их для дальнейшего предотвращения передачи малярии. Большая часть данных наблюдения будет иметь пространственные и временные признаки. С междисциплинарной точки зрения было бы интересно задать следующий важный и сложный вопрос: как построить более эффективный механизм наблюдения для мониторинга и раннего выявления относительной распространенности малярии и способов её передачи на основании имеющихся данных в пространственной и временной формах? Можно заметить, что существующие кластерные программные системы наблюдения не выявляют сети, лежащие в основе передачи малярии. Однако, изучение таких сетей может оказаться информативным и полезным, так как они характеризуют способы переноса малярии из одного места в другое. Они также могут помочь должностным лицам в здравоохранении и исследователям обнаружить скрытые и воздействующие друг на друга факторы, такие как внешняя среда, генетика и экология, и определить/предсказать схемы/тенденции передачи малярии. Сетевой подход является дальнейшим развитием существующих методов моделирования передачи малярии на основании набора выбранных факторов. В данной статье мы делаем обзор работ по выявлению сетей передачи, обсуждаем возможности применения такого подхода в

разработке эффективных вычислительных средств для выявления сетей передачи малярии на основании частичных данных наблюдения, какие методологические шаги могут быть предприняты и с какими проблемами придется столкнуться при создании и проверке этих средств.

Translated from English version into Russian by Natalia Potashnik, through

TRANSLATORS
WITHOUT BORDERS

Modelado de la transmisión de la malaria: una perspectiva de red

Liu Ji-ming, Yang Bo, William Cheung y Yang Guo-jing

Resumen

La transmisión de la malaria puede estar afectada por una multiplicidad de factores que incluso pueden no ser evidentes, lo que dificulta lograr una predicción precisa del impacto de los programas de eliminación y erradicación que se han implementado, y del posible resurgimiento y diseminación que puede continuar emergiendo. Un posible abordaje actual es el desarrollo y la implementación de sistemas de vigilancia a fin de identificarlos tan pronto como sea posible y así permitir que aquellos que desarrollan las políticas modifiquen e implementen estrategias para continuar previniendo la transmisión. La mayor parte de la información de vigilancia será de naturaleza espacial y temporal. Desde un punto de vista interdisciplinario, sería interesante plantearse la siguiente pregunta, que es tan importante como desafiante: sobre la base de la información de vigilancia disponible de forma espacial y temporal, ¿cómo podemos construir un mecanismo de vigilancia para controlar la prevalencia relativa y los patrones de transmisión de la malaria y lograr una detección temprana de ellos? Lo que podemos notar a partir de los sistemas de software de vigilancia basados en grupos es que no infieren las redes de transmisión subyacentes de la malaria. Sin embargo, dichas redes pueden brindar una gran cantidad de información y de detalles, ya que describen cómo se transmite la malaria de un lugar a otro. Estas redes, a su vez, pueden permitir a los responsables de las políticas de salud pública y a los investigadores en este campo descubrir los factores ocultos que interactúan, tales como el entorno, la genética y la ecología; y descubrir o predecir tendencias o patrones de transmisión de la malaria. La perspectiva de red extiende aún más los abordajes actuales del modelado de la transmisión de la malaria sobre la base de un conjunto de factores elegidos. En el presente artículo, sondeamos los trabajos relacionados sobre inferencia de las redes de transmisión, discutimos cómo puede utilizarse dicho abordaje para desarrollar un medio computacional

efectivo para deducir las redes de transmisión de la malaria sobre la base de datos de vigilancia parciales, y qué pasos metodológicos y qué problemas pueden estar involucrados en su formulación y validación.

Translated from English version into Spanish by Juan Manuel Macarlupu Peña, through

TRANSLATORS
WITHOUT BORDERS
